# Weekend new patient reviews in psychiatry: evaluation of activity over 3 months

**DOI:** 10.1192/pb.bp.117.056291

**Published:** 2017-12

**Authors:** Michael Rutherford, Mark Potter

**Affiliations:** 1 South West London and St George's Mental Health NHS Trust

## Abstract

**Aims and method** South West London and St George's Mental Health NHS Trust developed a system of weekend new patient reviews by higher trainees to provide senior medical input 7 days a week. To evaluate the effectiveness of these reviews, the notes for all patients admitted over 3 months were examined. The mean length of stay for patients before and after the introduction of the weekend new patient reviews were compared via unpaired *t*-test.

**Results** A total of 88 patients were seen: 84.4% of patients were seen within 24 h of admission. Higher trainees instituted some changes in 78.9% of patients. The most frequent action was to modify medication, in 47.8%. The average length of stay after the introduction of weekend reviews was not significantly different.

**Clinical implications** Weekend reviews of newly admitted patients by higher trainees is a feasible method for providing senior input to patients admitted out of hours.

Medical specialties of various types are increasingly being requested to deliver senior-led care throughout weekends and bank holidays. This 7-day working is felt to be potentially beneficial for patient care and has been started by many acute specialties. The Academy of Medical Royal Colleges released a report in 2013 outlining how this could be achieved for different specialties, including psychiatry.^1^ They recommended that 21–30% of psychiatric in-patients would benefit from a daily consultant-led review; mainly those who have been newly admitted or are clinically unstable. They stated that all new admissions, especially those under the Mental Health Act 1983, should receive a review the following day to initiate a treatment plan, including medication, observation levels, physical and psychosocial investigations and referrals.

The Academy of Medical Royal Colleges also advised that their recommendations were considered in light of the requirements of the local organisation as practice is likely to vary depending on geographic location, organisation size, on-call staffing and number of in-patient units. They also acknowledged that to achieve these outcomes, certain interventions and investigations would need to be available, such as pathology access.

## Method

South West London and St George's Mental Health NHS Trust developed a system of weekend ward reviews led by higher trainees with consultant advice. The higher trainees in the Trust were on a full-shift on-call rota with 09:00 h to 21:00 h shifts on weekends and bank holidays. Patients admitted out of hours on Fridays, Saturdays or up to 09:00 h on Sunday morning are reviewed by the on-call higher trainee the next day. Patients admitted after 09:00 h on Sunday are reviewed on Monday by the ward team, unless it is a bank holiday, in which case they are reviewed by the higher trainee on call that day. After the higher trainee has completed their reviews for a given site, they contact the on-call consultant to discuss the cases and ensure the management plans instituted are appropriate. This occasionally led to changes in treatment plan. This provides an opportunity for supervision and training and ensures consultant input. The role of the reviews was to ensure that the patients received prompt assessment by a senior clinician and a comprehensive management plan could be instituted. They were not intended to facilitate immediate discharge, although it was hoped that earlier treatment would reduce the time patients had to spend on the ward.

To evaluate the activity involved in the weekend new patient reviews, the notes for all patients admitted between 17:00 h on Friday and 09:00 h on Sunday between 1 January 2016 and 31 March 2016 were identified via the RiO system used by the Trust. This provided information regarding the timing of their admission. The clinical notes of each of these patients were examined to determine the reason for their admission, when they were seen by the on-call higher trainee, the diagnosis made and any actions taken as a result of the weekend review. The mean length of stay for patients admitted between 17:00 h on Friday and 09:00 h on Sunday and 1 January 2015 to 31 March 2015 were also compared via unpaired *t*-test. The number of serious untoward incidents in each time period was also compared via Mann–Whitney *U*-test.

## Results

A total of 88 patients were seen over the 3-month period. This equates to 6.8 patients appropriate for senior review each weekend.

Of those admitted, 46 (52.3%) were male. The average age was 41.9 years (range 18–84, s.d. = 17.8). For males the average age was 41.4 years (range 18–84, s.d. = 19.6) and for females it was 42.5 years (range 19–83, s.d. = 15.9). These differences were not significant on *t*-test.

The time from admission to senior review is indicated in Table 1. A total of 11 patients were not seen at all. Of these, 4 were admitted via the 136 Suite in the Trust.

**Table 1:** Time from admission to senior review

Review within	Number of patients	Cumulative number	Cumulative percentage of patients seen
12 hours	17	17	22.1

24 hours	48	65	84.4

36 hours	8	73	94.8

48 hours	4	77	100

Diagnoses were made by the higher trainee in 73 patients. The other 15 patients were diagnosed during later assessments. These diagnoses are shown in Table 2. There were no significant differences between patients diagnosed by the higher trainee or at a later point on two-tailed *z*-tests.

**Table 2:** Diagnoses made by higher trainees, the diagnoses of patients not seen or not diagnosed by the higher trainee

	Diagnosed by higher trainee		Diagnosed subsequently		Total diagnoses	
Diagnoses	Number of patients	Percentage of patients	Number of patients	Percentage of patients	Number of patients	Percentage of patients
Psychosis	31	42.5	8	53.3	39	44.3

Depression	17	23.3	7	46.7	24	27.2

Mania/hypomania	7	9.6	1	6.7	8	9.1

Alcohol or substance misuse	7	15.6	1	6.7	8	9.1

Personality disorder	12	2.6	3	20	15	17

Anxiety and stress-related disorders	2	1.3	2	13.3	4	4.5

Eating disorder	1	5.2	1	6.7	2	2.3

Developmental disorders	4	1	2	13.3	6	6.8

Physical illness	1	1.3	0	0	1	1.1

Unclear	1	1.3	1	6.7	2	2.3

The interventions made or recommended by higher trainees for the 77 patients reviewed during weekends are shown in Table 3. This does not include them indicating their agreement with actions made by clinicians who saw the patients before them. Again, the number of interventions shown exceeds the number of patients.

**Table 3:** Interventions made or recommended by higher trainees as a result of weekend new patient reviews

Intervention	Total, *n*	%
Start medication		
Regular antipsychotic	8	10.4
As required antipsychotic	2	2.6
Mood stabiliser	1	1.3
Antidepressant	8	10.4
Sedation	5	6.5
Hypnotic	3	3.9
Physical medication	3	3.9
Nicotine replacement	1	1.3
Total in which medication started	24	31.2

Increase medication		
Regular antipsychotic	1	1.3
Antidepressant	2	2.6
Sedation	3	3.9
Other psychotropics	1	1.3

Stop medication		
Antipsychotic	1	1.3
Sedation	1	1.3
Opiate replacement	1	1.3
All	2	2.6
Total medication changes	37	47.8

Recommended interventions		
Regular antipsychotic	2	2.6
Psychotherapy	3	3.9
Electroconvulsive therapy	1	1.3
Social interventions	1	1.3
Leave	1	1.3
Transfer	1	1.3
Other specific assessments	3	3.9

Discharge	2	2.6

Recommendation for Mental Health Act assessment	2	2.6

Physical investigations	8	10.4

Physical monitoring or treatments	8	10.4

Total physical health interventions	11	14.3

Transfer to psychiatric intensive care unit	3	3.9

Change in observations	3	3.9

Urine drug screen	3	3.9

Specific advice or information	3	3.9

Obtained collateral information	2	2.6

No action	17	22.1

The length of stay for patients admitted between Friday 17:00 h and Sunday 09:00 h between 1 January and 31 March in 2015 was 26 days (s.d. = 37) and for the same period of time in 2016 the length of stay was 28 days (s.d. = 43). This was not significant.

The number of serious untoward incidents between January and March 2015 was 13, and during the same period in 2016 it was 28; this was significant (*P* = 0.0652). However, when serious untoward incidents were limited to those occurring in acute services and out of hours, there was 1 incident between January and March 2015 and 3 incidents in the same time period in 2016; these numbers were too low to be analysed and so did not reach significance.

## Discussion

A substantial number of patients, 88 in total, were eligible for weekend review over the 3 months covered by the service evaluation and 77 of these were actually seen. A substantial minority of those not seen were admitted through the Trust's dedicated 136 Suite. This mode of admission may be a weak point in the current system, possibly as a patient detained to the suite could be viewed as having been admitted at that point, despite still awaiting formal assessment and possible admission. Patients admitted via the 136 Suite should still have been reviewed the following day.

Of those patients seen by the higher trainees, the vast majority (84.4%) were seen within 24 h and most of the rest, up to 94.8%, were seen within 36 h. Many of those seen between 24 and 36 h had been admitted during the morning on a Saturday and the higher trainee was unable to see them until the afternoon on Sunday. A small number (5.2%) were seen after 36 h had elapsed. The reasons for this are unclear but could be as a result of temporary limited provision of higher trainee cover due to illness.

The characteristics of the patients admitted do not appear to be remarkable, although it would have been useful to have a comparison group of patients admitted during the week to determine whether there were significant differences between the two, in terms of demographics, admission reason and diagnosis. It is unclear why some higher trainees did not formulate diagnoses for the patients they reviewed, but there appears to be no significant differences between those diagnosed during the weekend or following later assessments.

Higher trainees performed or recommended a wide variety of interventions for patients, instituting some changes in 78.9% of cases. The most frequent action was to start some form of medication; this was done for 31.2% of patients. If increasing and stopping medication is also considered, then medication changes were performed in 47.8% of cases seen by the higher trainees. These were in addition to prescriptions made by admitting core trainees.

The next largest group of interventions were recommended physical investigations and interventions, again, which had not been instituted on admission. This occurred in 14.3% of patients reviewed. These varied from instituting monitoring of fluid and food intake to obtaining specific investigations, such as lithium levels.

A variety of other interventions were performed. It is reassuring that only 2 patients needed to have a recommendation for detention under the Mental Health Act completed. The others appear to be appropriately informally or already detained under the Mental Health Act. The limited number of discharges would be at least partially explained by this not being the proposed aim of the weekend reviews.

In 22.1% of patients seen over the weekends no action was taken by the higher trainee. This could be due to all reasonable interventions already having been performed by an experienced core trainee or patients requiring a period of observation before any definitive management plans are made.

Despite these interventions being initiated earlier than expected, there was no change in the average length of stay for patients who were eligible for new patient reviews. This could be explained by the interventions only being delivered 24–48 h earlier than they otherwise would have been. The reviews were also not intended to facilitate immediate discharge. A greater focus on expediting discharge over the weekend may have led to a reduced length of stay.

Although the overall number of serious untoward incidents was significantly higher in 2016 than 2015, there was no significant difference when they were restricted to those associated with acute out-of-hours services which would appear most clearly related to the introduction of weekend new patient reviews. The overall increase in incidents may be due to a continuing Trust drive to improve reporting with a view to improving services, rather than a true increase and so the figures are difficult to interpret accurately in this context.

This system did not require any changes in rota patterns for higher trainees. The system was such that the reviews were carried out during the scheduled 09:00 h to 21:00 h shift, with reviews ceasing at 21:00 h so that patients could rest adequately without their evening or night being disrupted. On rare occasions when all the planned reviews could not be completed, they were postponed until the next day. Furthermore, as mentioned above, the focus of the reviews was not discharge; this prevented difficulties in coordinating with social care and other agencies during the weekend. The system used by the Trust enabled it to provide senior medical input 7 days a week in acute services without disrupting weekday working or leading to any of the other concerns raised by some authors.^2^

### Conclusions

There has been increasing emphasis on providing more senior weekend medical input across all specialties.^1^ In psychiatry, the recommendations were mainly in terms of newly admitted patients.

Weekend reviews of newly admitted patients by higher trainees, with consultant support, is a feasible and appropriate method for providing senior input to these patients who could potentially remain on a ward for more than 48 h without being seen by any clinician more senior than a core trainee. This would not be considered appropriate in any other medical specialty. If we are to be committed to parity of esteem in healthcare, then it is reasonable for patients admitted to psychiatric wards to be reviewed by a senior clinician within 24 h, as they would in any other hospital.^3^ This is particularly pertinent as the higher trainees provided some intervention in most patients, modifying medications in just under half of those admitted. It may be valuable to determine how this compares with patients reviewed during medical or surgical post-take rounds.

## References

[1] Academy of Medical Royal Colleges Seven Day Consultant Present Care: Implementation Considerations. Academy of Medical Royal Colleges, 2013.

[2] Keynejad R Holt C Rao R 7 day services and psychiatry. Lancet Psychiatry 2016; 3: 197–9. 26851877 10.1016/S2215-0366(16)00049-3

[3] Bailey S Thorpe L Smith G *Whole-Person Care: From Rhetoric to Reality. Achieving Parity between Mental and Physical Health* (Occasional Paper OP88). Royal College of Psychiatrists, 2013.

